# Microbial Biofilms in Urinary Tract Infections and Prostatitis: Etiology, Pathogenicity, and Combating strategies

**DOI:** 10.3390/pathogens5040065

**Published:** 2016-11-30

**Authors:** Cristina Delcaru, Ionela Alexandru, Paulina Podgoreanu, Mirela Grosu, Elisabeth Stavropoulos, Mariana Carmen Chifiriuc, Veronica Lazar

**Affiliations:** 1 Earth, Environmental and Life Sciences Section-ICUB, Research Institute of the University of Bucharest, University of Bucharest, Bucharest 060101, Romania; larioncristina@yahoo.com (C.D.); elisabeth.stavropoulos@yahoo.com (E.S.); veronica.lazar2009@gmail.com (V.L.); 2 Iancului Private Laboratory, Bucharest 060101, Romania; alexandru.ionela@lpiancului.ro (I.A.); podgoreanu.paulina@yahoo.com (P.P.); 3 Department of Microbiology & Immunology, Faculty of Biology, University of Bucharest, Bucharest 060101, Romania; mirela.grosu@yahoo.com

**Keywords:** UPEC 1, quorum sensing (QS) 2, antibiofilm 3

## Abstract

Urinary tract infections (UTIs) are one of the most important causes of morbidity and health care spending affecting persons of all ages. Bacterial biofilms play an important role in UTIs, responsible for persistent infections leading to recurrences and relapses. UTIs associated with microbial biofilms developed on catheters account for a high percentage of all nosocomial infections and are the most common source of Gram-negative bacteremia in hospitalized patients. The purpose of this mini-review is to present the role of microbial biofilms in the etiology of female UTI and different male prostatitis syndromes, their consequences, as well as the challenges for therapy.

## 1. Introduction

**Figure 1:**
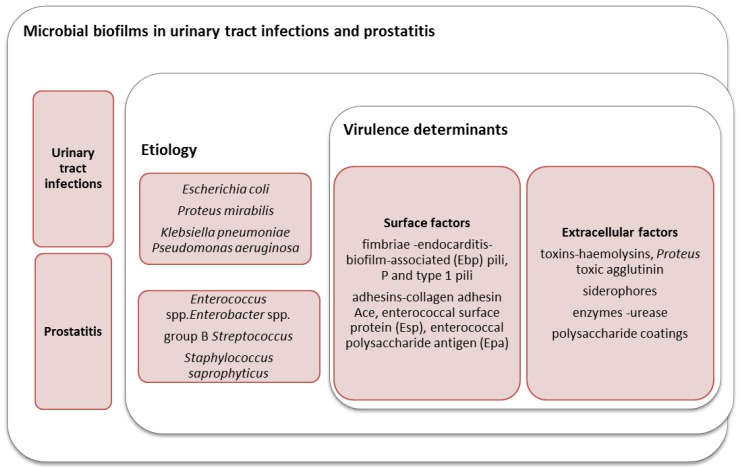
Etiology and virulence determinants of urinary tract microbial biofilms.

Urinary tract infections (UTIs) are among the most common bacterial infection in humans, occurring in either the community or healthcare setting. Due to high incidence, the financial implications of UTIs are enormous [1]. *Escherichia (E.) coli* is the most frequent agent (about 80%) of UTI in humans and one of the most common causes of Gram-negative bacteremia in hospitalized patients [2]. Other bacteria involved are *Proteus mirabilis*, *Klebsiella pneumoniae*, *Pseudomonas aeruginosa*, *Enterococcus* spp*.*, *Enterobacter* spp*.*, group B *Streptococcus*, and *Staphylococcus saprophyticus* [3]. The bacterial uropathogens harbor several virulence determinants necessary for initial adhesion and colonization of host mucosal surfaces, and for cell and tissue invasion, overcoming the host defense mechanisms, and causing persistent and chronic infections. Microbial virulence determinants include surface factors (fimbriae, adhesins, and P and type 1 pili) and extracellular factors (toxins, siderophores, enzymes, and polysaccharide coatings) [4,5,6] (Figure 1).

Microorganisms do not live as pure cultures of dispersed single cells but instead accumulate at interfaces to form polymicrobial aggregates such as films, mats, flocs, sludge, or “biofilms” [7]. Biofilms are microbial communities of surface-attached cells embedded in a self-produced extracellular polymeric matrix. They are the result of complex intra- and intercellular signaling and communication processes, regulated by a complex quorum sensing (QS) regulation system, which are ubiquitous in the microbial world [8]. The QS phenomenon is considered today as the mechanism that allows pathogenic bacteria to coordinate virulence factors expression for escaping the host immune response and establishing an infection [9]. Cell-to-cell communication or the QS mechanism has also been shown to be involved in biofilm development in the case of several uropathogens [10].

Biofilm development can be considered as a virulence determinant responsible for the long-lasting persistence of bacteria in the genitourinary tract [11]. Urinary catheters and any other prosthetic devices predispose to UTI by destroying natural barriers (urethral sphincter) and providing a nidus for infection by serving as a substrate for biofilm formation. Fundamental research studies have demonstrated that biofilm cells are more resistant to antimicrobial agents than planktonic bacterial cells [12]. Reduced antibiotic susceptibility of biofilms contributes to the persistence of infections, such as those associated with implanted devices. Poor antibiotic penetration, nutrients limitation, slow growth as an adaptive stress response, and the formation of persister cells are hypothesized to constitute multi-layered biofilm aspects [13].

Conventional antimicrobials are not effective against biofilms, and there are relatively few novel compounds or strategies under development or under clinical testing. Increased knowledge regarding the formation of biofilms has led to identification of several possible points for targeted antibiofilm approaches [7,14]. In the literature, synergistic interactions between different oil components and terpene–terpenoid combinations (e.g., carvacrol–α-pinene and carvacrol–myrcene) have been specified. Terpenes are substances with modest antimicrobial activity, favoring entry of terpenoids in the cell and manifestation of the antimicrobial effect by specific mechanisms. Moreover, synergistic action between aromatic terpenoids structure, i.e., eugenol–cinnamaldehyde, has been revealed, and both substances are known for their antimicrobial activity and antibiofilm formation [15]. Strategies to prevent the early onset of biofilm development involve a modification of abiotic and biotic surfaces, and a stimulation of the innate immune response [7,16].

### Medical Biofilms: Definition, Development Stages, and Properties

The definition of a biofilm has evolved over the years. Marshal in 1976 observed the presence of fine extracellular polymer fibrils that anchored bacteria to different surfaces [1]. A biofilm may be described as a microbial community characterized by cells that are attached to an interface, embedded in a matrix of exopolysaccharides, which demonstrates an altered phenotype [17]. Non-cellular materials, such as mineral crystals, corrosion particles, and blood components, depending on the environment in which the biofilm developed, may also be found in the biofilm matrix. Biofilm-associated organisms also differ from their planktonic (freely suspended) counterparts with respect to the genes that are transcribed [18]. 

Studies have shown that biofilm development begins immediately after catheter insertion; microorganisms attach to a conditioning film of host proteins associated with the catheter surface [8]. Initially, planktonic microbial cells are transported to the conditioned surfaces by physical forces or bacterial appendages, such as flagella. These first microbial colonists reversible adhere to the substrate through adsorption. Physical forces associated with bacterial adhesion include the van der Waals forces, and the steric and electrostatic (double-layer) interactions, collectively known as the DVLO (Derjaguin, Verwey, Landau, and Overbeek) forces [19]. Some reversibly adsorbed cells remain immobilized and become irreversibly adsorbed. Surface microbial structures, such as flagella, fimbriae, and pili overcome the physical repulsive forces of the electrical double layer. Subsequently, the appendages contact the conditioning layer stimulating chemical reactions and consolidating the bacteria–surface bond. It has been suggested that microbial adhesion strongly depends on the hydrophobic–hydrophilic properties of interacting surfaces [20]. Some bacteria are not able to attach to a surface on their own but can anchor themselves to the matrix or directly to earlier colonists, such as in the case of dental plaque. 

During the colonization process, microbial cells communicate via QS using signal molecules such as acyl-homoserin lactones (AHL) in the case of Gram-negative species and respectively, peptides in the case of Gram-positive bacteria [21]. Once the cells are firmly adhered to the surface, they start to aggregate through cell-to-cell interaction and produce an extracellular matrix composed of different types of biopolymers—known as extracellular polymeric substances (EPS’s)—which form the scaffold for the three-dimensional architecture of the biofilm and is responsible for the adhesion to surfaces and for the cohesion in the biofilm. The biofilm development determined for the microorganism a new lifestyle that is entirely different from the planktonic state [7]. The matrix serves as a nutrient reservoir and a protective barrier against adverse environmental conditions and stress (desiccation, biocides, antibiotics, and metallic cations, ultraviolet radiation, and host immune defense mechanisms) [22,23,24].

The biofilm matrix acts as a recycling center by keeping all the components of lysed cells available including DNA, which may represent a reservoir of genes for horizontal gene transfer [7]. Microbial cell detachment from mature biofilms occurs because of microbial enzymatic degradation of the extracellular matrix in response to environmental changes, i.e., nutrient limitation and oxygen depletion, or is mediated by external forces such as fluid shear, abrasion (collision of solid particles with the biofilm), predator grazing, and human intervention [25,26,27].

Biofilms pose a public health problem for individuals who require indwelling medical devices, as the microorganisms in the biofilms are difficult to treat with antimicrobial agents. The therapeutic efficacy of ciprofloxacin against UTIs including foreign body-associated UTIs caused by several uropathogens has been previously shown [28,29,30]. An experimental study based on the development of a catheter-based biofilm infection model in mice, using bioluminescent-engineered bacteria have proved to be an excellent model for evaluating antibacterial activity with chronic biofilm infections of the urinary tract. Induced UTIs in mice with bacterial cells in suspension were shown to respond rapidly to ciprofloxacin and were efficiently eradicated by this therapy. In contrast, despite the sensitivity of planktonic *P. mirabilis* Xen 44 and *P. aeruginosa* Xen 5 to ciprofloxacin, both pathogens induced a persistent and recurrent infection following the treatment of catheterized animals, demonstrating the difficulty of treating biofilm infections on implanted bodies [31].

Uropathogens differ in terms of the virulence factors and pathogenic mechanisms that allow them to colonize and infect the urinary tract. For example, *Proteus* spp. produces the enzyme urease, which hydrolyzes urea to ammonia and carbon dioxide. The release of ammonia raises the urinary pH, which favors the precipitation of urinary salts forming kidney or bladder stones, which frequently serve as a nidus for recurrent *P. mirabilis* infection [12]. Calcium crystals and magnesium ammonium phosphate precipitates are incorporated into polysaccharide microbial capsules, forming crystalline biofilms on the catheter [32]. *P. mirabilis* produces two toxins: hemolysin (HpmA), which destabilizes the host cell by inserting itself into the cell membrane, and *Proteus* toxic agglutinin (Pta), which produces holes in the host cell membrane, causing leakage of the cytosol, osmotic stress, and the depolymerization of actin filaments (Figure 1). Pta also induces bacterial cell-to-cell interaction via auto aggregation [33,34]. Enterococci also produce several adhesion factors involved in catheter-associated biofilm development, including the collagen adhesin Ace, the enterococcal surface protein (Esp), the enterococcal polysaccharide antigen (Epa), and the endocarditis- and biofilm-associated pili (Ebp) [35]. Urinary catheterization induces fibrinogen release into the bladder as part of the inflammatory response; this fibrinogen accumulates in the bladder and is further accumulated on the catheter. *E. faecalis* attaches to fibrinogen-coated catheters and uses it for growth, enhancing biofilm development on the catheter [32].

Biofilms are more resistant to antibiotics than planktonic cells, one of their hallmarks being their profound tolerance to a large spectrum of antimicrobials [36], due to several mechanisms, such as (i) the limitation of antibiotic diffusion through the matrix; (ii) the transmission of resistance genes within the community; (iii) the expression of efflux pumps and the inactivation of the antibiotics due to changes in metal ion concentrations and pH values; (iv) the physiological changes of microbial cells due to nutrient limitation environment (reduced metabolic and growth rates); (v) the presence of metabolically inactive cells known as persisters or dormant bacterial cells, as bacteria enter in a spore-like, non-dividing state, more tolerant to antimicrobials [23]; and (vi) the induction of a biofilm phenotype (expression of active mechanisms to combat the detrimental effects of antimicrobial agents) [37]. Increased knowledge regarding biofilm development has led to the identification of several possible points for targeted antibiofilm approaches [15]. Some strategies to prevent the early onset of biofilm development involve the modification of abiotic and biotic surfaces [15] and the stimulation of the innate immune response [16]. The use of substances that can destroy the physical integrity of the biofilm matrix is an attractive, antibiofilm approach, as the consequent loss of the highly protective barrier, represented by the exopolysaccharide matrix, exposes sessile microbial cells to antibiotics as well as to the innate host immune effectors [37].

## 2. The Role of Microbial Biofilms in the Etiology of UTIs in Women

The human vagina and the bacterial communities that reside therein represent a finely balanced mutualistic association [38]. The presence of *Lactobacillus* spp. is associated with a healthy state and is thought to protect reproductive-age women from non-indigenous pathogens, certainly by contributing to the maintenance of a low vaginal pH (<4.5) through the production of lactic acid. The vaginal microbiota is unique as it undergoes major compositional changes throughout a women’s lifespan from birth to puberty and menopause [39]. There has been an increasing recognition of the role of lactobacilli in the maintenance of the homeostasis within dynamic ecosystems such as the vagina and in the prevention of colonization and infection caused by pathogenic organisms.

Women are more prone to UTIs than men due to the proximity of the urethra, vagina, and rectum. The retrograde ascent of bacteria from the perineum is the most common cause of acute cystitis in women. Host factors such as changes in normal vaginal microbiota may also increase the risk of UTI in females. Postmenopausal women have a higher risk of UTI than younger women due to the lack of estrogen, which is essential to maintain normal vaginal fluid acidity. This acidity is necessary for normal growth of *Lactobacillus* sp., an important host defense mechanism against pathogenic organisms.

Acute UTIs caused by bacteria can turn into recurrent infections, which are defined as a “re-infection” when they involve a cause other than the initial infection, or are defined as a “relapse” when they are caused by the same strain as the strain originally involved in the UTI etiology [40]. Recurrent UTIs are usually new infections from bacteria outside the urinary tract (reinfection). The infection can be caused by the same or different organisms. Recurrent UTIs are common among young, healthy women. About 25% of women with an episode of acute cystitis develop recurrent UTI later [14]. The most common cause of UTIs in adult women is *E. coli*, followed by *Enterococcus faecalis*, *Klebsiella* spp., *Proteus* spp*.*, *Providencia* sp., *Morganella* sp., and *Staphylococcus (S.) saprophyticus*. However, in catheterized patients, the etiology is dominated by *Pseudomonas (Ps.) aeruginosa*, *Serratia*, *Enterobacter*, *Citrobacter*, and *Candida* species [41].

Uropathogenic *E. coli* (UPEC) possesses many redundant virulence factors that allow bacteria to resist and overcome various host defense mechanisms, namely, type 1 fimbriae and pili involved in adherence to host cells [42] and invasion; toxins and flagella that play an important role in pathogen dissemination, while different iron acquisition systems promote the survival of microorganisms in environments with iron low concentrations such as urinary tract. The immune response to UPEC is mediated mainly by toll-like receptors that recognize lipopolysaccharides, flagella, and other extracellular microbial structures. UPEC can undermine the host immune response by activating a pro-inflammatory response or by masking immunogenic structures [43]. Relapse due to UPEC was related to the ability of these pathogenic strains to form biofilms. In such cases, biofilm development may be crucial for UPEC persistence in the vagina and/or bladder, being related to persistence and recurrence [44].

After *E. coli*, *Proteus* sp. strains (particularly *P. mirabilis*) are the most frequent causative agents of UTI especially in the case of patients with low immunity. This species possesses several virulence factors, including fimbriae (mannose resistant/Proteus-like fimbriae (MR/P), adherence to urinary epithelium cells/non-agglutinant fimbriae (UCA/NAF) and *P. mirabilis* fimbriae (PMF)), flagella, antigenic variation, capsule, IgA protease, LPS, and metabolic enzymes (protease, urease, and hemolysins), hydroxyapatite crystal formation, and iron acquisition systems [45]. After initial attachment, *P. mirabilis* multiply and form biofilms that protect it from the host immune response and antibiotics. The gallstones favor the formation of biofilms during the UTIs associated with *P. mirabilis*.

Most catheterized patients with recurrent urinary infections caused by *P. mirabilis* (62%) develop bladder stones [46,47]. Increased urinary pH causes local supersaturation and the precipitation of calcium phosphate and magnesium phosphate-ammonium and form crystals of apatite and struvite [48]. In catheterized patients, the urease producing micro-organisms, such as *P. mirabilis*, *P. vulgaris*, and *Providencia rettgeri* induce a pH increase followed by the formation of ammonium ions, leading ultimately to the precipitation of magnesium phosphate and bi/tricalcium phosphate crystals [49]. These crystals may form a protective layer against the anti-microbial effects of newly surface modified catheters [50,51].

Enterococci have become an increasingly common cause of UTI, representing more than 30% of all bacterial isolates causing UTI [42]. Enterococci are intrinsically resistant to many antimicrobials and may develop resistance to a range of antibiotics [52]. The presence of enterococci in the urinary tract is often asymptomatic [53]. Unfortunately, the treatment of UTIs often involves the use of broad-spectrum antibiotics, which are a risk factor for the development of strains resistant to vancomycin (VRE) [54]. Despite the limited utility of pyuria in the diagnosis of enterococcal UTI, a more than threefold increase in cases of the inappropriate use of antibiotics was observed, suggesting a need for an accurate diagnosis of urinary infections [55,56]. The diversification of bacterial biofilm has many consequences for bacterial survival and productivity, and this is key to developing new antimicrobial strategies and diagnostic techniques. Additionally, diversification can improve biofilm productivity because of the more efficient use of available resources [57].

## 3. The Role of Biofilms in Prostatitis and Urethritis in Men

*S. epidermidis*, enterococci, and diphtheroids are found frequently in the anterior urethra of males, while *E. coli*, *Proteus* sp., and nonpathogenic *Neisseria (N.)* species are occasionally reported. The normal microbiota residing in the urethra must be taken into consideration in the clinical interpretation of urine cultures [57]. Uroepithelium adherent bacteria may invade the renal tissue causing pyelonephritis and chronic bacterial prostatitis. Moreover, prostatitis may be difficult to diagnose because the colonized bacteria may not be present in prosthetic secretion or urine samples [58]. The etiology of acute bacterial prostatitis and chronic bacterial prostatitis, defined as a persistent bacterial infection of the prostate lasting more than three months, is akin to that of acute UTIs. Sexually active men younger than 35 years and older men who engage in high-risk sexual behaviors need to be tested for *N. gonorrhoeae* and *Chlamidia trachomatis* [57]. A great percentage of *E. coli* strains collected from patients with prostatitis exhibit the ability to develop biofilms, which may explain the difficulty in treating of infections. Acute bacterial prostatitis is most commonly caused by ascending UTI produced by *E. coli*, *P. mirabilis*, *Ps. aeruginosa*, *Klebsiella* spp., *Enterococcus* spp., and *Serratia* spp. [59]. The biofilm may develop on the epithelium, urinary calculi, prostate, and implanted foreign devices [60,61]. In the host cell, they form an intracellular bacterial community (IBC) with biofilm-like properties, firstly described in UPEC, associated with chronic cystitis and recurrent UTIs in children. The bacteria adherent to urinary epithelium develop biofilms and can invade the renal tissue causing pyelonephritis or chronic bacterial prostatitis with sterile urine culture. 

Macrolides (erythromycin, clarithromycin, and azithromycin) are the first-choice antibiotics having “in vitro” and “in vivo” high antibiofilm activity [62]. Clinicians should attempt to make an accurate diagnosis of UTIs, as this will be crucial in the choice of the appropriate narrow-spectrum antibiotics [63] and thus in the prevention of antibiotic resistance emergence [64].

## 4. Catheter Associated Infections

The risk of developing a UTI significantly increases with the use of indwelling devices. Catheter-associated UTI (CAUTI) is one of the most common care-associated infections around the world, accounting for around 80% of all nosocomial UTIs, all patients becoming colonized by Day 30 [65]. The environmental conditions created on the catheter surface make it an ideal site for bacterial attachment and formation of biofilm structures [66]. In this type of medical device, urease-producing microorganisms may cause encrustation, formation of infected bladder calculi, and urinary obstruction. The crystal layer protects bacteria from the antimicrobial effects of compounds used for coating or impregnating the catheters. In addition, biofilm formation may even result in the increased ability of uropathogens to cause acute prostatitis and persist in the prostatic secretory system, leading to recurrent UTIs characteristic to chronic bacterial prostatitis [1].

## 5. Antimicrobial Strategies for Fighting Against Urinary Tract Biofilms

The failure or restricted penetration of antimicrobial agents into biofilms, the entrance in slow-growing or starvation states, the selection of persisters, or other stress tolerant phenotypes are known to be antibiotic tolerance mechanisms of biofilms [67] that result in infections that are difficult to treat and require complex multi-drug treatment strategies, especially when biofilms are polymicrobial [68].

### 5.1. Antibiotics

The effectiveness of antibiotics belonging to different classes to penetrate biofilm matrix varies. For example, cationic aminoglycosides are trapped by the negatively charged polymers of the biofilm matrix, the beta-lactams, and glycopeptides diffusion is reduced, while the fluoroquinolones and rifampicin penetrate immediately, explaining the rapid installation of the bactericidal effect (19 min) [69], as well as macrolides [70]. The most efficient antibiofilm combinations cited in the literature are clarithromycin plus vancomycin, and roxithromycin plus imipenem [36]. To reduce the side effects of antibiotics, co-administration of probiotics is necessary to restore intestinal homeostasis after prolonged treatment with antibiotics or immunological imbalances, which is achieved by reducing the production of pro-inflammatory cytokines and the prevention of epithelial cells apoptosis [71,72].

### 5.2. Natural Antimicrobial Compounds

Plants can synthesize many molecules with antibiofilm activity, such as *Ibicella corpora lutea*, *Grandis coccinea* extracts, volatile oils from *Rosmarinus officinali*s and *Salvia officinalis*, *Mentha piperita, Eugenia caryophyllata*, ylang ylang, vanilla, patchouli, *Satureja hortensis*, and lichens [73,74]. Plant extracts also exhibit the advantage of reduced side effects. In addition, plant extracts have a pronounced anti-microbial activity at sub-inhibitory concentrations, which do not interfere with microbial growth, but only with their behavior, therefore not selecting resistance [75]. Other natural compounds with antimicrobial activity against biofilms are probiotic compounds, such as acids and antimicrobial peptides [76].

### 5.3. Nanoparticles

Metal cations and related compounds exhibit important antimicrobial features, depending on the nanoparticles shape, size, concentration, contact time as well as the different physico-chemical conditions of the environment. By their small size, nanoparticles can penetrate bacterial cells, disrupt cellular membranes, and bind to chromosomal DNA [1,77]. The remarkable antimicrobial features of the nanostructured metal ions and their compounds could represent promising solutions for preventing and combating harmful biofilms not only in medical, but also in ecological and industrial fields [14,78].

### 5.4. Antimicrobial Coatings

The nanotechnology is also used to obtain new biomaterials more resistant to microbial colonization or as antimicrobial drug release systems or carriers [79]. Different antimicrobial agents were used for coating and impregnation of urinary catheters such as antibiotics (nitrofurazone, gentamicin, norfloxacin, and ciprofloxacin), silver, synthetic cationic peptides, bacteriophages expressing an enzyme involved in the degradation of the biofilm (efficiency of about 99.9%), gendine (violet gentian plus chlorhexidine), nitrous oxide, nitrofurazone (nitrofuran), minocycline + rifampicin, hydrogels, nanoparticles MgF fluoride, ytrium (YF3), CaO, and antagonistic nonpathogenic bacteria. However, the risk of developing antibiotic resistance when antibiotic levels become sub inhibitory is very high [1,80,81].

### 5.5. Enzyme Inhibitors

Urease-producing bacteria are known to produce crystalline biofilms and encrustation on catheters. The urease inhibitors have also been used to prevent urea breakdown via the in vitro increase of *P. mirabilis* pH to decrease the associated encrustation [1,82]. Urease inhibitors are represented by plant extract compounds such as fluorofamide, vanillic acid, prune juice, and γ-lactones germanica, by bilberry extracts with anti-adhesin proprieties, by low surface acoustic waves, and by diguanilat cyclase inhibitors [83,84].

Studies have showed that the crystalline layer of biofilms developed on urinary catheters is composed of two main types of crystals: struvite and apatite. Bacteria are in intimate association with this crystal layer and protect them from the antimicrobial effects of impregnated compounds. Therefore, avoidance of urinary pH decrease and subsequent crystallization could be critical in preventing biofilm development on indwelling devices [84]. 

### 5.6. Bacteriophages

Another antimicrobial approach for the prevention of biofilm-associated catheter development is bacteriophages. Catheters coated with T4 bacteriophages active against *E. coli* and coli-proteus bacteriophages active against *Proteus* have been developed. It was observed that the phage treatment of catheters led to an approximately 90% reduction in biofilm formation compared to control catheters [1].

### 5.7. Quorum Sensing Inhibitors

Quorum sensing (QS) inhibitors are a potential tool for UTI-associated biofilm control. Furanones have been shown to interfere with *S. epidermidis* biofilm development on urinary catheters in animal models [43], and azithromycin inhibited QS-dependent phenotypes *in vitro*. Recent studies revealed that several natural compounds exhibit antimicrobial properties through the modulation of QS phenotypes [21].

### 5.8. Other Antibiofilm Strategies

Other antibiofilm strategies are represented by iontophoresis (i.e., the improvement of antibiofilm drug efficiency via an application of a low-intensity electric field) and the use of liposomes as carriers or delivery vehicles for active hydrophilic and hydrophobic molecules. Using nonpathogenic bacteria (known as bacterial interference or antagonisms) for colonization of the catheters surface can prevent adhesion and biofilm formation by pathogens [1].

Ideal antibiofilm therapy is potentially represented by a compound or group of compounds that exhibit not only antibiofilm activity but also the ability to interfere with the expression of virulence factors.

## 6. Conclusions

Biofilms constitute an important contribution to the high incidence, recurrence, and complications of UTIs, thus requiring efficient prevention and control measures. Biofilm research will lead to a better understanding of the disease process and will subsequently lead to the development of new prevention and treatment options. An ideal approach will include a combination of antibiofilm molecules, with an anti-pathogenic effect, active at sub-inhibitory concentrations to reduce the risk of developing resistance and with low toxicity for the host cells.
